# Association of Childhood Violence Exposure With Adolescent Neural Network Density

**DOI:** 10.1001/jamanetworkopen.2020.17850

**Published:** 2020-09-23

**Authors:** Leigh G. Goetschius, Tyler C. Hein, Sara S. McLanahan, Jeanne Brooks-Gunn, Vonnie C. McLoyd, Hailey L. Dotterer, Nestor Lopez-Duran, Colter Mitchell, Luke W. Hyde, Christopher S. Monk, Adriene M. Beltz

**Affiliations:** 1 Department of Psychology, University of Michigan, Ann Arbor; 2 Serious Mental Illness Treatment Resource and Evaluation Center, Office of Mental Health and Suicide Prevention, Department of Veterans Affairs, Ann Arbor, Michigan; 3 Department of Sociology, Princeton University, Princeton, New Jersey; 4 Teachers College & College of Physicians and Surgeons, Columbia University, New York, New York; 5 Survey Research Center of the Institute for Social Research, University of Michigan, Ann Arbor; 6 Population Studies Center of the Institute for Social Research, University of Michigan, Ann Arbor; 7 Neuroscience Graduate Program, University of Michigan, Ann Arbor; 8 Department of Psychiatry, University of Michigan, Ann Arbor

## Abstract

**Importance:**

Adverse childhood experiences are a public health issue with negative sequelae that persist throughout life. Current theories suggest that adverse childhood experiences reflect underlying dimensions (eg, violence exposure and social deprivation) with distinct neural mechanisms; however, research findings have been inconsistent, likely owing to variability in how the environment interacts with the brain.

**Objective:**

To examine whether dimensional exposure to childhood adversity is associated with person-specific patterns in adolescent resting-state functional connectivity (rsFC), defined as synchronized activity across brain regions when not engaged in a task.

**Design, Setting, and Participants:**

A sparse network approach in a large sample with substantial representation of understudied, underserved African American youth was used to conduct an observational, population-based longitudinal cohort study. A total of 183 adolescents aged 15 to 17 years from Detroit, Michigan; Toledo, Ohio; and Chicago, Illinois, who participated in the Fragile Families and Child Wellbeing Study were eligible for inclusion. Environmental data from birth to adolescence were collected via telephone and in-person interviews, and neuroimaging data collected at a university lab. The study was conducted from February 1, 1998, to April 26, 2017, and data analysis was performed from January 3, 2019, to May 22, 2020.

**Exposures:**

Composite variables representing violence exposure and social deprivation created from primary caregiver reports on children at ages 3, 5, and 9 years.

**Main Outcomes and Measures:**

Resting-state functional connectivity person-specific network metrics (data-driven subgroup membership, density, and node degree) focused on connectivity among a priori regions of interest in 2 resting-state networks (salience network and default mode) assessed with functional magnetic resonance imaging.

**Results:**

Of the 183 eligible adolescents, 175 individuals (98 girls [56%]) were included in the analysis; mean (SD) age was 15.88 (0.53) years and 127 participants (73%) were African American. Adolescents with high violence exposure were 3.06 times more likely (95% CI, 1.17-8.92) to be in a subgroup characterized by high heterogeneity (few shared connections) and low network density (sparsity). Childhood violence exposure, but not social deprivation, was associated with reduced rsFC density (β = −0.25; 95% CI, −0.41 to −0.05; *P* = .005), with fewer salience network connections (β = −0.26; 95% CI, −0.43 to −0.08; *P* = .005) and salience network-default mode connections (β = −0.20; 95% CI, −0.38 to −0.03; *P* = .02). Violence exposure was associated with node degree of right anterior insula (β = −0.29; 95% CI, −0.47 to −0.12; *P* = .001) and left inferior parietal lobule (β = −0.26; 95% CI, −0.44 to −0.09; *P* = .003).

**Conclusions and Relevance:**

The findings of this study suggest that childhood violence exposure is associated with adolescent neural network sparsity. A community-detection algorithm, blinded to child adversity, grouped youth exposed to heightened violence based only on patterns of rsFC. The findings may have implications for understanding how dimensions of adverse childhood experiences impact individualized neural development.

## Key Points

**Question:**

Are violence exposure and social deprivation associated with person-specific patterns (heterogeneity) of adolescent resting-state functional connectivity?

**Findings:**

In this cohort study of 175 adolescents, childhood violence exposure, but not social deprivation, was associated with reduced adolescent resting-state density of the salience and default mode networks. A data-driven algorithm, blinded to childhood adversity, identified youth with heightened violence exposure based on resting-state connectivity patterns.

**Meaning:**

Childhood violence exposure appears to be associated with adolescent functional connectivity heterogeneity, which may reflect person-specific neural plasticity and should be considered in neuroscience-based interventions.

## Teaser

This cohort study examines functional connectivity in adolescents who were exposed to violence during childhood.

## Introduction

Adversity during childhood is a common, detrimental public health issue. Adverse childhood experiences negatively impact physical and mental health, and effects likely persist into adulthood.^1,2,3^ Early adverse environments have underlying dimensions, such as violence exposure (eg, neighborhood violence) and social deprivation (eg, neglect),^4,5^ which have distinct neural correlates related to emotion, fear, and reward processing.^4,6^ For instance, violence exposure and social deprivation are associated with blunted amygdala and ventral striatum reactivity, respectively.^5^ However, it is unclear how these dimensions affect neural circuitry. Adverse childhood experiences not measured dimensionally are associated with alterations in resting-state functional connectivity (rsFC) of the salience network (SN), which is a task-positive network including the anterior insula that is involved in identifying and integrating salient input,^7^ and the default mode network (DMN), which is a task-negative network including the inferior parietal lobule that is linked to internal thought, memory, and social-cognitive processes.^8,9,10,11^ However, inferences are limited by relatively small, homogeneous samples focused on few brain regions using retrospective reports of adversity.^12^ Thus, there are significant knowledge gaps concerning the ways in which early violence exposure and social deprivation impact later functioning of neural circuits and how that impact varies across individuals.

Neural circuits are typically studied using a network framework, with key features including density (ie, number of connections^13^) and node degree (ie, number of connections involving a specific brain region).^14,15^ Across development, network density increases between distal nodes and node degree increases for hub regions, such as the anterior insula.^16^ Understanding how early adversity relates to network density has a potential for revealing how the environment affects brain development.^14^

These influences are likely to be person specific because there is considerable variability in neural responses to environmental stress,^17^ and thus, mean-based analyses may not accurately reflect an individual’s circuitry.^18^ Data from neuroimaging projects, such as the Midnight Scan Club, have illustrated that the organization of an individual’s rsFC is unique and qualitatively different from the group average.^19^ Moreover, consistent with behavioral studies of early adversity,^20^ average estimates of adversity’s effects on the brain often have high variances.^21,22,23^ This variance raises the question of whether there is information about individual differences and their causes—important for eventual prevention and intervention—that is not being conveyed by mean-based conclusions.

In the present study, we examined the association between dimensional indexes of childhood exposure and individualized adolescent rsFC networks. We used a large, longitudinal sample of adolescents with a substantial representation of African American and low-income participants, who are often underrepresented in neuroimaging research,^12^ and a person-specific rsFC approach that detects meaningful connections among brain regions while identifying subgroups of participants that share network features (group iterative multiple model estimation [GIMME]).^24,25^ We hypothesized that childhood violence exposure and social deprivation would be associated with person-specific indexes of SN and DMN density, respectively. This study was preregistered with the Center for Open Science.

## Methods

Participants were from the Fragile Families and Child Wellbeing Study (FFCWS), a population-based cohort study of children born in large US cities, with an oversample of nonmarital births as well as a large proportion of families of racial/ethnic minorities with low resources.^26^ This study followed the Strengthening the Reporting of Observational Studies in Epidemiology (STROBE) reporting guideline for cohort studies. This study was approved by the University of Michigan Institutional Review Board. Caregivers and participants provided informed consent or assent. Participants received financial compensation.

In the FFCWS, data were collected from the primary caregiver (94% biological mothers) and focal child at their birth and at ages 1, 3, 5, 9, and 15 years through in-home visits and phone calls.^27^ Data for the violence exposure and social deprivation composites, which have been previously reported,^5,6,28^ came from surveys collected at ages 3, 5, and 9 years. During wave 6 (when the focal child was approximately age 15 years), 237 adolescents from Detroit, Michigan; Toledo, Ohio; and Chicago, Illinois, participated in a supplementary visit during which rsFC data were collected. The study was conducted from February 1, 1998, to April 26, 2017, and data analysis was performed from January 3, 2019, to May 22, 2020. To our knowledge, rsFC data and their association with early adversity have not been previously published. Owing to the representative sampling of the FFCWS, youth were not preselected based on their willingness to participate in a magnetic resonance imaging (MRI) study, which is a common procedure in neuroimaging. This sampling approach led to missing or incomplete MRI data (n = 54) but was not statistically significantly different from the recruited sample (eMethods and eTable 1 in the Supplement).

### Participants and Procedure

A total of 183 adolescents aged 15 to 17 years were eligible for inclusion. The participants and their primary caregiver came to a university lab and completed questionnaires regarding the focal child’s current life stress, pubertal development, and other demographic variables. During an MRI scan, 8 minutes of data were collected while the adolescents were instructed to remain still and focus on a white fixation cross on a black screen.

Violence exposure and social deprivation were each operationalized by composite *z* scores calculated from FFCWS data at ages 3, 5, and 9 years; thus, 0 is the approximate mean. Both constructs included primary caregiver’s report of experiences that directly (eg, physical abuse) and indirectly (eg, community support) affect the child (eMethods in the Supplement). Violence exposure was operationalized as physical or emotional abuse directed at the child, exposure to intimate partner violence, and witnessing or being victimized by community violence. Social deprivation was operationalized as emotional or physical neglect, lack of romantic partner support for the mother, and lack of neighborhood cohesion.^5^ To reflect a comprehensive assessment of cumulative, dimensional childhood exposure to violence and social deprivation, both constructs included experiences with several levels of proximity to the child (eg, home and neighborhood) at multiple ages.^5,29^

To address potential confounding factors, sensitivity analyses adjusted for sex, race, pubertal development,^30^ adolescent life stress,^31,32,33^ maternal educational level at the child’s birth, and maternal marital status at the child’s birth^26^ (eMethods in the Supplement).

### Neuroimaging Measures

#### MR Acquisition

Magnetic resonance data were acquired using a 3T scanner with an 8-channel head coil (GE Discovery MR750, GE Healthcare). Head movement was minimized through instructions to the participant and padding was placed around the head. Functional T2*-weighted blood oxygenation level–dependent images were acquired using a reverse spiral sequence^34^ of 40 contiguous axial 3-mm slices (eMethods in the Supplement).

#### Imaging Data Analysis

Preprocessing was primarily conducted in FSL, version 5.0.7.^35,36^ Structural images were skull-stripped and segmented. Functional images were skull-stripped, spatially smoothed, registered to subject-specific structural and Montreal Neurological Institute space, and corrected for motion using MCFLIRT^37^ and ICA-AROMA.^38^ Nuisance signal from white matter and cerebrospinal fluid was removed. Data were high-pass filtered. As an additional precaution against motion-related artifacts, participants with an average relative framewise displacement greater than 0.5 mm before motion preprocessing were excluded (n = 4) (eMethods in the Supplement).

Participant-specific time series (235 functional volumes) from 7 regions of interest (ROIs) per hemisphere (14 total) were extracted. The ROIs and their locations were selected using Neurosynth^39^ and preregistered. Four bilateral ROIs defined the SN: amygdala, insula, dorsal anterior cingulate cortex, and dorsal prefrontal cortex. Three bilateral ROIs defined the DMN: inferior parietal lobule (IPL), posterior cingulate cortex, and medial temporal gyrus. Regions of interest were 6.5-mm spheres around central coordinates (eTable 2 in the Supplement) linearly adjusted for participant brain volume.

#### GIMME

Subgrouping GIMME (S-GIMME, version 0.5.1)^24,25,40^ in R, version 3.5.1 (The R Project for Statistical Computing) was used for rsFC analyses (eFigure 1 in the Supplement). Beginning with an empty network, S-GIMME fits person-specific unified structural equation models^25^ in a data-driven manner by using Lagrange multiplier tests^41^ to add directed connections among ROIs that are contemporaneous (occurring at the same functional volume) or lagged (occurring at the previous volume, including autoregressives) and that apply to the group level (everyone in the sample), the subgroup level (everyone in a data-derived subsample), or individual level (unique to an individual). S-GIMME is a sparse mapping approach in which only connections that account for a significant amount of variance are added to each participant’s network until the model fits the data well according to standard fit indexes (root-mean-square error of approximation ≤0.05; standard root mean residual ≤0.05; confirmatory fit index ≥0.95; and nonnormed fit index ≥0.95).^42,43^ During model generation, connections that have become nonsignificant with the addition of new connections are pruned. S-GIMME uses a community detection algorithm (Walktrap) to detect subgroups of participants and determine their shared connectivity patterns. S-GIMME has been described and validated in large-scale simulations and applied to empirical data.^40,42,44,45^ Network density and node degree of only the contemporaneous connections were extracted from person-specific S-GIMME networks, because lagged connections control for sequential dependencies (eg, hemodynamic response function).^25,46^

### Statistical Analysis

Inferential analyses were completed in R, version 3.5.1 and examined whether childhood adversity statistically significantly predicted adolescent neural network features. First, binary logistic regression was used to statistically predict S-GIMME–detected subgroup membership from childhood violence exposure and social deprivation. Second, multiple regression was used to statistically predict density (ie, number of connections) within the SN, within the DMN, and between the SN and DMN from childhood violence exposure and social deprivation. Third, multiple regression was used to statistically predict node degree (ie, number of connections involving a specified node) for each of the 14 ROIs from violence exposure and social deprivation using a Bonferroni-corrected significance threshold (*P* < .004). In follow-up sensitivity analyses, covariates were added to all regressions to assess the robustness of observed effects.

## Results

**Figure 1.**
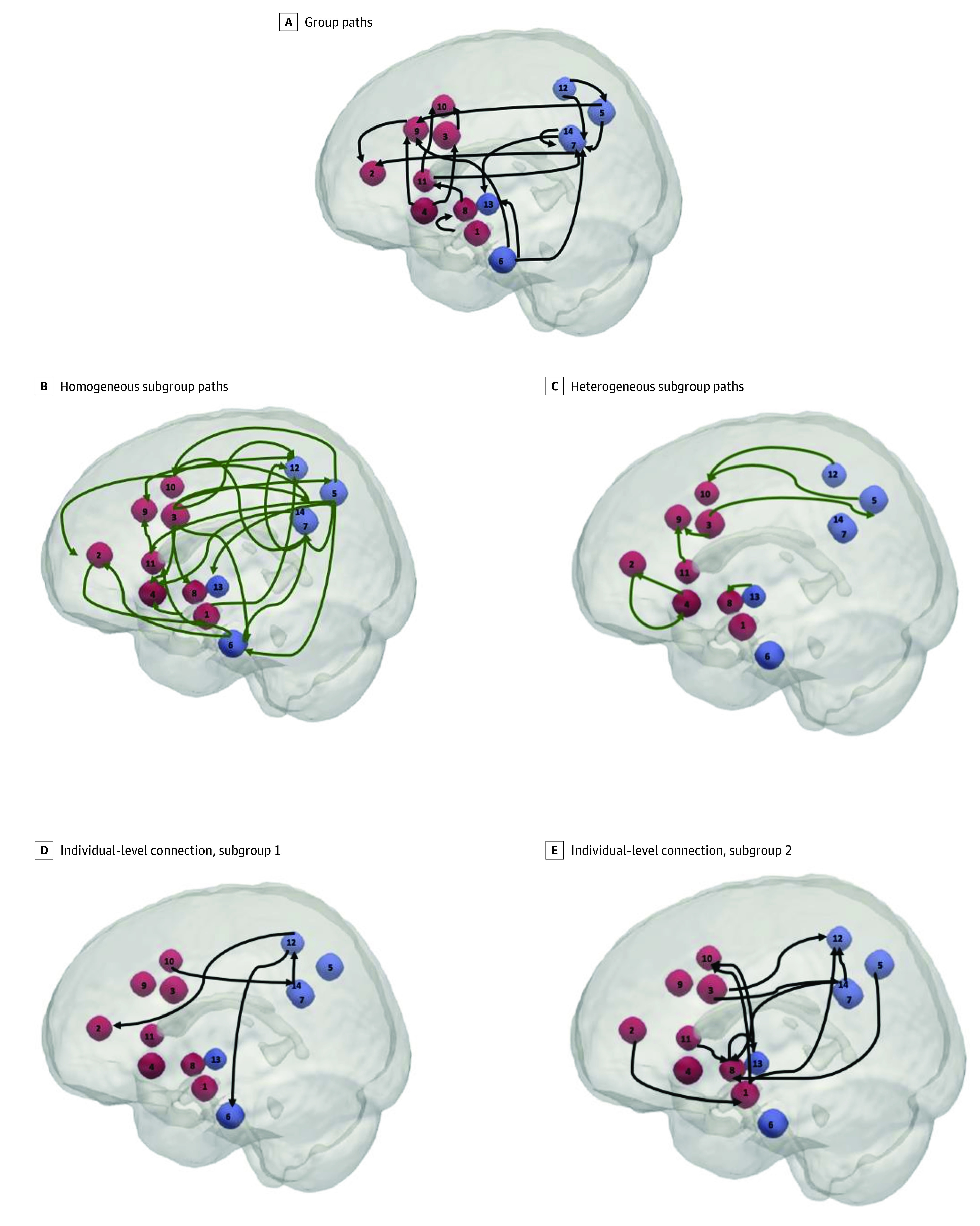
S-GIMME Connectivity Results A, Connections fit at the group-level (ie, statistically meaningful for ≥75% of sample) (n = 175). B, Subgroup-level connections for first algorithm-detected subgroup (n = 42). C, Subgroup-level connections for second algorithm-detected subgroup (n = 133). D, Individual-level connections for illustrative participant in first subgroup. E, Individual-level connections for illustrative participant in second subgroup. All connections are directed and contemporaneous. Red nodes are part of the salience network. Blue nodes are part of the default mode network.

Of the 183 participants, 175 adolescents aged 15 to 17 years (mean [SD] age, 15.88 [0.53] years) were included in the analysis. Of the 183 SAND adolescents with resting-state MRI data, 3 were excluded owing to artifacts in the functional or structural MRI data, 4 had excessive motion (as defined by average relative framewise displacement >0.5 mm), and 1 had signal dropout in the areas of the brain included in the present analysis. Of these 175 adolescents, 98 participants (56%) were girls, 77 participants (44%) were boys, and 127 participants (73%) were African American. All person-specific resting state networks fit the data well, according to average indexes: root-mean-square error of approximation = 0.06, standard root mean residual = 0.05, confirmatory fit index = 0.93, and nonnormed fit index = 0.96 (individual indexes in eTable 7 in the Supplement). Group-level connections were detected within and between the SN and DMN (Figure 1A), 2 subgroups of participants with subgroup-level connections were identified (Figure 1B, C), and all person-specific maps contained individual-level connections (mean [SD], 11.61 [5.32]) (eFigure 2 in the Supplement and the Video show individual maps). Final maps revealed that the first subgroup (n = 42) (Figure 1B, D) was qualitatively homogeneous with 27 subgroup-level connections and few individual-level connections (mean [SD], 5.6 [03.19]), and that the second subgroup (n = 133) Figure 1C, E) was qualitatively heterogeneous, with 8 subgroup-level connections and many individual-level connections (mean [SD], 13.50 [4.36]). Possible extreme outliers or overfit models did not impact results (eMethods in the Supplement).

**Video.** Individual Model Maps

Violence exposure was associated with subgroup membership (b = 1.12, *P* = .03). With a unit increase in violence exposure, participants were 3.06 (95% CI, 1.17-8.92) times more likely to be classified in the larger, heterogeneous subgroup (Table 1). In sensitivity analyses, the odds ratio was 2.54 (eTable 6 in the Supplement). Members of the heterogeneous subgroup experienced higher levels of childhood violence exposure (mean [SD], 0.09 [0.53]) than those in the homogeneous subgroup (mean [SD], −0.15 [0.43]). Social deprivation was not associated with subgroup membership (Table 1).

**Table 1.** Logistic Regression Results for Association Between Violence Exposure and Social Deprivation and Subgroup Membership While Controlling for Motion^a^

Variable	b (SE)	Odds ratio (95% CI)
Intercept	0.55 (0.32)	1.73 (0.90-3.22)
Violence exposure^b^	1.12 (0.52)	3.06 (1.17- 8.92)
Social deprivation	−0.49 (0.46)	0.61 (0.25-1.54)
Motion^b^^,^^c^	7.96 (3.59)	2860.05 (6.33- 8 236 598)

^a^ Heterogeneous or homogeneous subgroup membership.

^b^ Significant factor associated with subgroup membership.

^c^ Motion indicated by mean relative framewise displacement.

**Figure 2.**
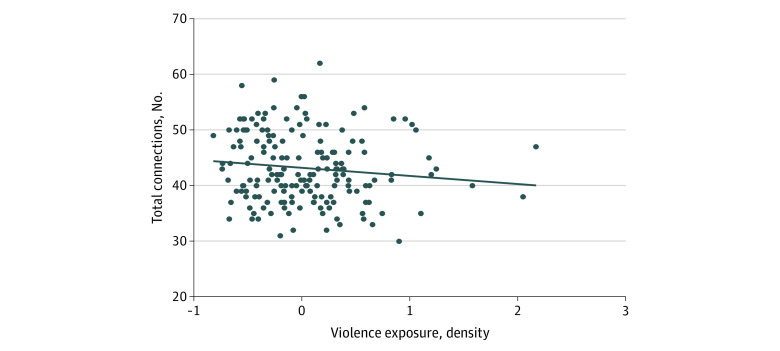
Association Between Childhood Violence Exposure and Reduced Network Density Number of connections modeled for each child. Adjusted *R*^2^ = 0.059.

Childhood violence exposure was related to reduced density (ie, sparsity) in the person-specific maps (β = −0.25; 95% CI, −0.41 to −0.05; *P* = .005; adjusted *R*^2^ = 0.059) (Figure 2). Specifically, violence exposure was associated with sparsity within the SN (β = −0.26; 95% CI, −0.43 to −0.08; *P* = .005) and between the SN and DMN (β = −0.20; 95% CI, −0.38 to −0.03; *P* = .02) (Table 2), including in sensitivity analyses (eTable 3 in the Supplement). Violence exposure was not associated with DMN density, and social deprivation was not related to network metrics (Table 2).

**Table 2.** Regression Results for Association Between Dimensional Exposure to Adversity and Network Density and Node Degree^a^^,^^b^

Variable	b (95% CI)^c^	β (95% CI)^d^	*r*^e^	Fit
**Total density**				
Intercept	41.88 (40.57 to 43.19)			*R^2^* = 0.075; 95% CI, 0.01-0.15
Violence exposure^f^	−3.08 (−5.20 to −0.96)	−0.25 (−0.41 to −0.05)	−0.12	
Social deprivation	1.81 (−0.28 to 3.91)	0.15 (−0.05 to 0.30)	.06	
Motion^f^^,^^g^	12.98 (3.55 to 22.40)	0.21 (0.06 to 0.37)	0.17^f^	
**Salience network density**				
Intercept	15.92 (15.47 to 16.37)			*R^2^* = 0.088; 95% CI, 0.02-0.16
Violence exposure^f^	−1.09 (−1.82 to −0.36)	−0.26 (−0.43 to −0.08)	−0.11	
Social deprivation	0.65 (−0.08 to 1.37)	0.15 (−0.02 to 0.32)	0.07	
Motion^f^^,^^g^	5.17 (1.91 to 8.43)	0.24 (0.09 to 0.39)	0.20	
**Density between salience and default mode networks**				
Intercept	16.03 (15.32 to 16.74)			*R^2^* = 0.079; 95% CI, 0.01-0.15
Violence exposure^f^	−1.33 (−2.48 to −0.19)	−0.20 (−0.38 to −0.03)	−0.07	
Social deprivation	0.76 (−0.37 to 1.90)	0.12 (−0.06 to 0.29)	0.06	
Motion^f^^,^^g^	8.58 (3.48 to 13.67)	0.25 (0.10 to 0.40)	0.23	
**Default mode network density**				
Intercept	9.93 (9.50 to 10.36)			*R^2^* = 0.024; 95% CI, 0.00-0.07
Violence exposure	−0.66 (−1.35 to 0.03)	−0.17 (−0.35 to 0.01)	−0.13	
Social deprivation	0.40 (−0.28 to 1.09)	0.10 (−0.07 to 0.28)	0	
Motion^a^	−0.77 (−3.85 to 2.30)	−0.04 (−0.19 to 0.12)	−0.06	
**Left inferior parietal lobule degree**				
Intercept	7.33 (6.98 to 7.68)			*R^2^* = 0.050; 95% CI, 0.00-0.11
Violence exposure^h^	−0.85 (−1.41 to −0.28)	−0.26 (−0.44 to −0.09)	−0.17	
Social deprivation	0.49 (−0.07 to 1.05)	0.15 (−0.02 to 0.33)	0.03	
Motion^g^	1.16 (−1.35 to 3.68)	0.07 (−0.08 to 0.22)	0.03	
**Right insula degree**				
Intercept	7.72 (7.48 to 7.96)			*R^2^* = 0.067; 95% CI, 0.01-0.14
Violence exposure^h^	−0.65 (−1.04 to −0.26)	−0.29 (−0.47 to −0.12)	−0.16	
Social deprivation	0.42 (0.03 to 0.80)	0.19 (0.01 to 0.36)	0.06	
Motion^g^	1.38 (−0.36 to 3.12)	0.12 (−0.03 to 0.27)	0.08	

^a^ Significant findings on nodes are reported here; results for all nodes are in eTable 4 in the Supplement.

^b^ Network density is the number of connections modeled by network; node degree, the sum of the modeled connections involving each node.

^c^ Unstandardized regression weights.

^d^ Standardized regression weights.

^e^ Zero-order correlation.

^f^ Significantly associated with network density at *P* < .05.

^g^ Motion indicated by mean relative framewise displacement prior to motion correction.

^h^ Indicates significant association with node degree at a Bonferroni-corrected *P* value <.05 divided by 14 (the number of statistical comparisons) to get the new Bonferroni-corrected *P* threshold nodes (*P* < .004).

Childhood violence exposure was related to reduced node degree for the right insula (β = −0.29; 95% CI, −0.47 to −0.12; *P* = .001) and left IPL (β = −0.26; 95% CI, −0.44 to −0.09; *P* = .003) using a Bonferroni-corrected significance threshold (Table 2; eTable 4 in the Supplement), including in sensitivity analyses (eTable 5 in the Supplement). There were no significant associations with social deprivation.

## Discussion

Results from a predominantly understudied and underserved sample with high rates of poverty suggest that childhood violence exposure, but not social deprivation, is associated with adolescent neural circuitry. Data-driven analyses identified a subset of adolescents with heterogeneous patterns of connectivity (ie, few shared and many individual connections) in 2 key neural networks associated with salience detection, attention, and social-cognitive processes (ie, the SN and DMN).^7,8^ This subgroup of adolescents was exposed to more violence in childhood than the other subgroup, whose patterns of neural connectivity were relatively more homogeneous (ie, had many connections in common), suggesting that violence exposure may lead to more person-specific alterations in neural circuitry. Beyond subgroups, network density within the SN and between the SN and DMN was sparse for adolescents with high violence exposure, likely due to few connections involving the right insula and the left IPL. These factors could not be accounted for by social deprivation, in-scanner motion, race, sex, pubertal development, current life stress, or maternal marital status or educational level at the time of the participant’s birth.

Findings regarding the neural network subgroups are noteworthy because the community detection algorithm within GIMME detected rsFC patterns in the brain from exposures that occurred at least 6 years earlier. Moreover, high childhood violence exposure in the subgroup characterized by neural heterogeneity likely reflects the person-specific outcomes of early adversity on the brain and suggests that research on the developmental sequelae of adverse childhood experiences should consider individual differences in neural compensatory responses to stress.^17^ Although it is important to replicate these findings in other samples, S-GIMME has reliably classified subgroups in empirical data,^40,45^ and there is evidence from simulations that modeling connections at the subgroup level, in addition to the group level, improves the validity and reliability of results.^40^

Considering the sample as a whole, results also suggest that violence exposure is associated with blunted connectivity within the SN and between the SN and DMN. As expected, the observed reduced SN density in adolescents with heightened childhood violence exposure differs from typical developmental patterns that show stronger rsFC within SN nodes and increased density of connections with hub regions, such as the anterior insula, as the brain matures.^8,16^ It is difficult, however, to align the present findings with previous work that reported increased SN rsFC in trauma-exposed youth^9,10^ because those samples were small, used different metrics of connectivity, and had different sample compositions. Moreover, the present sample was likely experiencing chronic adversity, and research from animal models of chronic stress proposes that, over time, the body’s stress response (eg, hypothalamic pituitary adrenal axis reactivity) becomes blunted or habituated to typical stressors.^47^ Previous research on hypothalamic pituitary adrenal axis reactivity in this sample revealed a blunted cortisol response in adolescents with heightened childhood violence exposure,^28^ and work in other high-risk samples showed blunted activation of the amygdala, an SN node, to threatening stimuli.^48,49^ The present study expands this notion to the function of threat detection neural circuits, and future research should examine whether this is compensatory or even adaptive.

Beyond density, childhood violence exposure was associated with reduced node degree of the right anterior insula and left IPL. These results are consistent with the extant literature because the right anterior insula in the SN facilitates shifting between the DMN and central executive network,^50^ which contributes to higher-level executive function.^8^ Moreover, early life stress has been linked to insular connectivity within the SN,^9^ DMN (specifically, the left IPL, which plays a role in working memory^51^), and other neural ROIs.^52^ These results also show differences in the way that the anterior insula is integrated within and between neural networks in youth exposed to violence in their homes and neighborhoods using longitudinal data from a population-based sample.

This study represents a person-specific approach to the neuroscientific investigation of the sequelae of early adversity. Past research on early adversity and rsFC assumed that the same connectivity patterns characterize all, or a majority of participants, but if this assumption is violated (as is likely the case in studies of diverse populations and biopsychosocial phenomena), then results may not accurately describe any individual.^18,53^ The presence of group- and subgroup-level connections in the present study suggests that there is some consistency in the connections within and between the SN and DMN, aligning with an assumption of homogeneity that is prevalent in rsFC research, but the large number of individual-level connections, especially in adolescents with high levels of early violence exposure, show that there was also notable heterogeneity that required person-specific analyses to accurately reflect rsFC, encouraging future research using person-specific modeling approaches.

All significant findings concerned violence exposure, and there were no detected associations between social deprivation and rsFC. This set of results could indicate that social deprivation has a less salient influence on patterns of spontaneous neural fluctuations. Some studies have identified links between social deprivation and functional connectivity, but they concerned extreme, nonnormative deprivation (eg, previous institutionalization).^21,54^ This deprivation may be qualitatively different from deprivation operationalized in the present study, and may operate through different mechanisms. In addition, because a hypothesis-driven approach to node selection was taken in this study, it is possible that deprivation is associated with rsFC of SN or DMN nodes not measured here, with other networks (eg, central executive), or in different populations (eg, with extreme or heightened variability of deprivation). It is also tenable that there are other dimensions of adversity that would have differential associations with rsFC (eg, those linked to emotionality), which future research should explore. Nonetheless, these findings present evidence for dimensional frameworks of adversity^5,55^ because there were distinct neural correlates for violence exposure.

### Limitations

This study had limitations. Based on the demographic characteristics of the sample (eg, 73% African American, born in Midwestern cities), it is not clear whether findings will generalize beyond low-income, urban, African American youth; nonetheless, the present work is important because these populations are often underrepresented in neuroimaging research and underserved by the medical community.^12^ Resting-state functional MRI was collected on only a single occasion in adolescence; thus, it is unclear whether connectivity patterns reflect stable or changing neural features. In addition, it is not possible to know the direction of association (eg, whether neural differences predate exposure to adversity). Violence exposure and social deprivation composites were derived from parent reports. Exposures between the FFCWS collection waves at ages 9 and 15 years could not be accounted for in this study. Owing to changes in the FFCWS questionnaire at year 15, current adversity could not be controlled using the composite scores created for earlier ages.^5^ To compensate, a life stress scale was used as a covariate; however, that confounding variable did not impact associations. The ecologic pattern of poverty-related adversity is complex; thus, there are unmeasured variables that may explain these associations or contribute to cascades of risk (eg, parental psychopathologic factors).

## Conclusions

In this prospective, longitudinal study, childhood violence exposure, but not social deprivation, was associated with person-specific differences in how the adolescent brain functions in regions involved in salience detection and higher-level cognitive processes. These differences were potent enough that a data-driven algorithm, blinded to child adversity, grouped youth with heightened violence exposure based on the heterogeneity of their neural networks, suggesting that the impact of violence exposure may have divergent and personalized associations with functional neural architecture. These findings have implications for understanding how dimensions of adversity affect brain development, which may inform future neuroscience-based policy interventions.

## Notes

Supplement. **eMethods.** Detailed Methods eReferences **eTable 1.** Participant Demographics **eTable 2.** MNI Coordinates for ROIs **eTable 3.** Regression Results for Network Density **eTable 4.** Node Degree for ROIs That Were Not Significantly Associated With Violence Exposure or Social Deprivation Using the Bonferroni-Corrected Significance Threshold **eTable 5.** Node Density Adjusted for Covariates **eTable 6.** Logistic Regression Results Adjusted for Covariates **eFigure 1.** Flowchart of the S-GIMME Analytical Steps **eFigure 2.** Individual Connectivity Maps **eTable 7.** Model Fit for Each Participant
