# Error in Subject Area

**DOI:** 10.1001/jamanetworkopen.2020.31600

**Published:** 2020-12-07

In the Original Investigation titled “Association of Childhood Violence Exposure With Adolescent Neural Network Density,”^1^ published September 23, 2020, there was an error in the subject area. On publication, the subject area listing that follows the Original Investigation heading was not visible. The subject area should have been “Pediatrics.” This article has been corrected.^1^
