# Supplementary material for: Association of Childhood Violence Exposure With Adolescent Neural Network Density
Source: JAMA Netw Open. 2020 Sep 23;3(9):e2017850. doi: 10.1001/jamanetworkopen.2020.17850 (PMC7512058; doi:10.1001/jamanetworkopen.2020.17850)
Supplement: Supplement. — eMethods. Detailed Methods eReferences eTable 1. Participant Demographics eTable 2. MNI Coordinates for ROIs eTable 3. Regression Results for Network Density eTable 4. Node Degree for ROIs That Were Not Significantly Associated With Violence Exposure or Social Deprivation Using the Bonferroni-Corrected Significance Threshold eTable 5. Node Density Adjusted for Covariates eTable 6. Logistic Regression Results Adjusted for Covariates eFigure 1. Flowchart of the S-GIMME Analytical Steps eFigure 2. Individual Connectivity Maps eTable 7. Model Fit for Each Participant [file jamanetwopen-e2017850-s001.pdf]

Supplementary Online Content

Goetschius LG, Hein TC, McLanahan SS, et al. Association of childhood violence exposure with adolescent neural network density. *JAMA Netw Open*. 2020;3(9):e2017850. doi:10.1001/jamanetworkopen.2020.17850

eMethods. Detailed Methods

eReferences

eTable 1. Participant Demographics

eTable 2. MNI Coordinates for ROIs

eTable 3. Regression Results for Network Density

eTable 4. Node Degree for ROIs That Were Not Significantly Associated With Violence Exposure or Social Deprivation Using the Bonferroni-Corrected Significance Threshold

eTable 5. Node Density Adjusted for Covariates

eTable 6. Logistic Regression Results Adjusted for Covariates

eFigure 1. Flowchart of the S-GIMME Analytical Steps

eFigure 2. Individual Connectivity Maps

eTable 7. Model Fit for Each Participant

This supplementary material has been provided by the authors to give readers additional information about their work.

© 2020 Goetschius LG et al. *JAMA Network Open.*

eMethods. Detailed Methods

Supplemental Participant Information

Five hundred and six families from Detroit, Toledo, and Chicago area who had participated in the FFCWS were contacted. Of the 506 FFCWS families contacted, 237 families participated in SAND data collection, and 183 of those families had teens who were eligible and completed the neuroimaging tasks. 54 teens either were not eligible (i.e., braces, etc.) to complete the neuroimaging portion of the study (N=28) or did not complete the full protocol (N=26).

Families that agreed to participate in SAND data collection did not differ from families that refused or were unreachable on annual household income at the six waves of the FFCWS (ps = .11 - .84). However, nonparticipation was associated with mother reports of community violence exposure when adolescents were ages 3, 5, and 9 ($\chi 2 = 6.72$, $df = 1$, $p < .05$, $V = .12$). Sixty-three percent of all mothers from families that participated in SAND endorsed at least one form of community violence when adolescents were ages 3, 5, or 9 compared to 51.10% of families that did not participate. Within the Detroit, Toledo, and Chicago subsamples, mothers' self-report of race/ethnicity at the time of the child's birth did not differ between mothers who did and did not participate in SAND (ps = .13-.49). However, mothers' self-report of race/ethnicity at the time of the child's birth differed between the SAND sample and the larger FFCWS ($\chi 2 = 86.32$, $df = 3$, $p < .01$, $V = .13$). The majority of mothers who participated in SAND data collection identified as Black/African American (75%), and also included mothers identifying as White/Caucasian (16%), Hispanic (6%), and other (3%). In the FFCWS, 46% of mothers identified as Black/African, 21% identified as White/Caucasian, 29% identified as Hispanic, and 4% as other.

Of the 183 SAND teens with resting-state MRI data, three participants were excluded due to artifacts in the functional or structural MRI data, four participants had excessive motion (as defined by average relative framewise displacement greater than 0.5mm), and one person had signal dropout in the areas of the brain included in the present analysis. The sample included for the neuroimaging analyses did not differ from the full SAND sample (*ps* 0.670 - 0.997) (Supplemental Table 1). This information has also been reported in other work from our group[1,2].

MRI Acquisition

Structural MRI Acquisition. T1-weighted gradient echo images were taken before the functional scans using the same field of view (FOV) and slices as the functional scans (TR = 12ms, TE = 5ms, TI = 500ms, flip angle = 15°, FOV = 26cm; slice thickness = 1.4mm; 256 x 192 matrix; 110 slices, voxel size = 1mm x 1mm x 1mm). These methods are described in previous research using this sample[3].

Resting State MRI Acquisition. Slices were prescribed parallel to the AC-PC line (same locations as structural scans). Images were reconstructed into a 64x64 matrix (TR=2000 ms, TE=30 ms, flip angle=90°, FOV=22 cm, voxel size=3.44mm x 3.44mm x 3mm, bottom-up interleaved). Slices were acquired contiguously, which optimized the effectiveness of the movement post-processing algorithms. Images were reconstructed off-line using processing steps to remove distortions caused by magnetic field inhomogeneity and other sources of misalignment to the structural data, which yields excellent coverage of subcortical areas of interest. These methods are identical to those described in previous task-based fMRI research using this sample[3].

© 2020 Goetschius LG et al. *JAMA Network Open.*

Imaging Data Analysis

MRI Preprocessing. Anatomical images were homogeneity-corrected using SPM8, then skull-stripped (f=0.25) using the Brain Extraction Tool (BET) in FSL (version 5.0.7)[4,5]. The functional imaging data then had the following preprocessing steps applied: removal of large temporal spikes in k-space data (> 2 std dev), field map correction and image reconstruction using custom code in MATLAB; noise from cardiac and respiratory motion were removed using RETROICOR, and slice-timing correction using SPM8 (Wellcome Department of Cognitive Neurology, London, UK; http://www.fil.ion.ucl.ac.uk). Additionally, the first 10 volumes of functional data were removed to ensure the stability of signal intensity. Lastly, the structural images were segmented into gray matter, white matter, and CSF using FSL's FAST[6].

Following these initial preprocessing steps, the resting state functional data underwent further preprocessing using FEAT (FMRI Expert Analysis Tool) Version 6.00, part of FSL (FMRIB's Software Library, www.fmrib.ox.ac.uk/fsl). Registration to high resolution structural and/or standard space images was carried out using FLIRT[7,8]. The following pre-statistics processing was applied: motion correction using MCFLIRT[8]; non-brain removal for the functional images using BET[4]; spatial smoothing using a Gaussian kernel of FWHM 6.0mm; grand-mean intensity normalization of the entire 4D dataset by a single multiplicative factor. ICA-AROMA[9] was used to remove motion-related artifacts in the data. Nuisance signal derived from the white matter and cerebrospinal fluid (CSF) were regressed out of the data and then the data was high-pass filtered to remove signal below 0.01Hz.

Violence Exposure and Social Deprivation Composite Scores

The procedure creating these composite scores were first utilized in previous work from our lab[2] and has been used previously in research on cortisol reactivity in this sample[10]. Data for these composite scores come from primary caregiver report on survey measures at 3, 5, 9 years.

Childhood Exposure to Violence. Included in this composite was the primary caregiver's report of child physical and emotional abuse based on items from the Parent-Child Conflict Tactics Scale[11] that have been used in previous research[12,13]. Five items were used to assess physical abuse including, "hit him/her on the bottom with a hard object" and "shook him/her" and five items were used to assess emotional abuse including whether the parent/caregiver has "sworn or cursed at," or "called him/her dumb or lazy or some other name like that." Each item was rated on a 7-point Likert scale ranging from "never happened" to "more than 20 times." The primary caregiver's report of the child's exposure to or victimization of violence in the neighborhood[14] was also included in the composite. This was measured using the primary caregiver's report of the child witnessing or being the victim of beating, attacks with a weapon, shootings, and killings (witness only) on a 5-point Likert scale ranging from "never" to "more than 10 times." At age 9, the primary caregiver was not asked about whether the child had witnessed killings or it they had been the victim of a shooting, so these items were only included for ages 3 and 5 years. Lastly, the child's mother reported on intimate partner violence (IPV) (physical-2 items, emotional-3 items, or sexual-1 item) in the home at each wave[13]. Each item was rated on a 3-point Likert scale ranging from "never" to "often." Physical IPV items included "he slapped or kicked you" and "he hit you with his fist or a dangerous object." Emotional IPV items included "he tried to isolate you from family and friends," and "he tried to

© 2020 Goetschius LG et al. *JAMA Network Open.*

prevent you from going to work and/or school." The sexual IPV was "he tried to make you have sex or do sexual things you didn't want to do." The child's exposure to IPV against the mother was coded as missing for a given wave if the child did not live with their mother at least 50% of the time. We considered violence exposure to exist on a continuum in which high scores represent violence exposure and low scores represent safety.

Childhood Exposure to Social Deprivation. Included in this composite was the primary caregiver's report of child physical and emotional neglect based on items from the CTS-PC[11] that have been used in previous research[12,13]. Four items from the CTS-PC were used to assed physical neglect including whether the parent was ever "so drunk or high that you had a problem taking care of your child." One item, whether the parent was "ever so caught up in your own problems that you were not able to show or tell your child that you loved him/her," was used to asses emotional neglect. These items from the CTS-PC were reported on the same 7-point Likert scale as the items in the violence exposure composite. The primary caregiver's report of social cohesion in the neighborhood was also included in this composite (reverse coded such that higher scores corresponded to lower cohesion). The items were selected based on previous research in the FFCWS linking neighborhood cohesion and adolescent mental health[15] and were adapted from previous neighborhood research[16]. This included 4 items, such as "this is a close-knit neighborhood," rated on a 5-point Likert scale ranging from "strongly agree" to "strongly disagree." Lastly, the child's mother reported on the level of intimate partner support for each wave using six items, such as "how frequently (the current romantic partner) expresses love and affection (for the mother)," that were rated on a 3-point Likert scale ranging from "never" to "often"[17]. This was also reverse coded such that high scores represent low support. Child exposure to the mother's intimate partner support was coded as missing for a given wave if the

child did not live with their mother at least 50% of the time. We considered social deprivation to exist on a continuum in which high scores (e.g., the child experienced either high neglect or witnessed low social support for their mother or low neighborhood social cohesion) represent deprivation and low scores (e.g., the child experienced little neglect or witnessed high social support for their mother or high neighborhood social cohesion) represent social support.

Composite Score Calculation. To calculate composite scores, the Z scores for each of the childhood experiences (i.e., child abuse, exposure to intimate partner violence, community violence, child neglect, lack of romantic partner support, lack of neighborhood social cohesion) were summed for each of the childhood experiences within a dimension (i.e., violence exposure and social deprivation)[18] and then divided by the number of childhood experiences within a dimension for each participant, thus maximizing the number of participants and the diversity of the sample by minimizing drop out due to missing data at any given wave. This procedure has been previously described[2].

In an exploratory attempt to characterize the sample, we examined the abuse and neglect subscales of the Conflict Tactics Scale (CTS). We found that averaged across ages 3, 5, and 9 years, the focal children experienced greater than 3 of the 10 abuse categories (*M*=3.67, *SD*=1.61, range: 0-10) and less than 1 of the 5 neglect categories (*M*=0.19, *SD*=0.37, range: 0-4) in the CTS scale.

© 2020 Goetschius LG et al. *JAMA Network Open.*

Covariates

With the exception of mean framewise displacement, all covariates have been previously described[2,3,10].

Current Life Stress. Current life stress was measured using the Adolescent Life Events Scale (adapted[19] from[20,21]). This scale assesses the experience of common adolescent stressful life events in the past year.

Race. Race was operationalized as a set of two dummy-coded variables based on three race categories reported by the teen: African American (Hispanic and Non-Hispanic), White/Caucasian, and Other. Other included Hispanic, Asian, multi-racial participants, Native American, and Unknown/Not Reported (Table 1). If teen report was not available ($N = 12$), then parent report was used.

Pubertal Development. Self-report of pubertal status was assessed using child report of the Pubertal Development Scale[22] that measures growth spurt in height, public hair, and skin change in boys and girls; facial hair growth and voice change in boys only; and breast development and menarche in girls only (Table 1). When adolescent report was not available (N=8), parent report was used. Pubertal development scores reported by parents were not significantly different from those reported by the adolescent ($t(9.04)=-0.74$, $p=0.48$).

Maternal Covariates at Birth. Maternal self-report of marital status at birth (yes/no) and education at birth (1 - less than high school, 2 - high school or equivalent, 3 - some college/technical school, 4 - college or graduate school) was assessed.

Framewise Displacement. Framewise displacement (FD) is a metric that evaluates motion in the scanner and is the average of rotation and translation parameter differences[23]. FD was quantified using fsl_motion_outliers (FSL v.5.0.7).

Sensitivity Analyses

Extreme Outliers. Predictor variables (i.e., violence exposure and social deprivation) were checked for extreme outliers, which were considered to be individuals with a value less than Q1 - 2.2*Interquartile Range (IQR) or greater than Q3 + 2.2*IQR[24]. Four individuals were considered to be extreme outliers. Analyses were run with and without these participants, and inferences did not change. Thus, the participants were not excluded in order to retain as much data as possible.

Protecting Against Model Overfitting. To protect against model overfitting, the psi matrices for each individual were examined – these matrices summarize the variance that was not explained for each network node for each participant. Participants were flagged (*N*=26 or 14.8%) if they had a psi value greater than 1. To ensure that these participants were not driving effects, all analyses were run with and without participants with high psi values, and inferences did not change. Thus, the participants were not excluded in order to retain as much data as possible.

© 2020 Goetschius LG et al. *JAMA Network Open.*

eReferences

1. Peckins MK, Roberts AG, Hein TC, et al. Violence exposure and social deprivation is associated with cortisol reactivity in urban adolescents. *Psychoneuroendocrinology*. Published online September 4, 2019:104426. doi:10.1016/j.psyneuen.2019.104426

2. Hein TC. Dimensions of Early Adversity as Distinct Predictors of Adolescent Brain Development. Published online 2019. https://deepblue.lib.umich.edu/bitstream/handle/2027.42/149945/heint_1.pdf?sequence=1&isAllowed=y

3. Goetschius LG, Hein TC, Mattson WI, et al. Amygdala-prefrontal cortex white matter tracts are widespread, variable and implicated in amygdala modulation in adolescents. *NeuroImage*. 2019;191:278-291.

4. Smith SM. Fast robust automated brain extraction. *Hum Brain Mapp*. 2002;17(3):143–155.

5. Jenkinson M, Beckmann CF, Behrens TEJ, Woolrich MW, Smith SM. FSL. *NeuroImage*. 2012;62(2):782-790. doi:10.1016/j.neuroimage.2011.09.015

6. Zhang Y, Brady M, Smith S. Segmentation of brain MR images through a hidden Markov random field model and the expectation-maximization algorithm. *IEEE Trans Med Imaging*. 2001;20(1):45-57. doi:10.1109/42.906424

7. Jenkinson M, Smith S. A global optimisation method for robust affine registration of brain images. *Med Image Anal*. 2001;5(2):143-156.

8. Jenkinson M, Bannister P, Brady M, Smith S. Improved optimization for the robust and accurate linear registration and motion correction of brain images. *NeuroImage*. 2002;17(2):825-841.

9. Pruim RHR, Mennes M, van Rooij D, Llera A, Buitelaar JK, Beckmann CF. ICA-AROMA: A robust ICA-based strategy for removing motion artifacts from fMRI data. *NeuroImage*. 2015;112(Supplement C):267-277. doi:10.1016/j.neuroimage.2015.02.064

10. Peckins M, Roberts A, Hein T, et al. Early stress, deprivation, and threat as predictors of cortisol, DHEA, and cortisol/DHEA ratios in adolescence. *Psychoneuroendocrinology*. 2019;100:S62. doi:10.1016/j.psyneuen.2018.12.210

11. Straus MA, Hamby SL, Finkelhor D, Moore DW, Runyan D. Identification of Child Maltreatment With the Parent-Child Conflict Tactics Scales: Development and Psychometric Data for a National Sample of American Parents. *Child Abuse Negl*. 1998;22(4):249-270. doi:10.1016/S0145-2134(97)00174-9

12. Font SA, Berger LM. Child Maltreatment and Children's Developmental Trajectories in Early- to Middle-Childhood. *Child Dev*. 2015;86(2):536-556. doi:10.1111/cdev.12322

© 2020 Goetschius LG et al. *JAMA Network Open.*

13. Hunt TKA, Slack KS, Berger LM. Adverse childhood experiences and behavioral problems in middle childhood. *Child Abuse Negl*. 2017;67:391-402. doi:10.1016/j.chiabu.2016.11.005

14. Zhang S, Anderson SG. Low-income single mothers' community violence exposure and aggressive parenting practices. *Child Youth Serv Rev*. 2010;32(6):889-895. doi:10.1016/j.childyouth.2010.02.010

15. Donnelly L, McLanahan S, Brooks-Gunn J, et al. Cohesive Neighborhoods Where Social Expectations Are Shared May Have Positive Impact On Adolescent Mental Health. *Health Aff (Millwood)*. 2016;35(11):2083-2091. doi:10.1377/hlthaff.2016.0721

16. Morenoff J, Sampson R, Raudenbush S. Neighborhood Inequality, Collective Efficacy, and the Spatial Dynamics of Urban Violence. *Criminology*. 2001;39:517-560.

17. Manuel JI, Martinson ML, Bledsoe-Mansori SE, Bellamy JL. The influence of stress and social support on depressive symptoms in mothers with young children. *Soc Sci Med*. 2012;75(11):2013-2020. doi:10.1016/j.socscimed.2012.07.034

18. Song M-K, Lin F-C, Ward SE, Fine JP. Composite variables: when and how. *Nurs Res*. 2013;62(1):45-49. doi:10.1097/NNR.0b013e3182741948

19. Shaw DS, Gilliom M, Ingoldsby EM, Nagin DS. Trajectories leading to school-age conduct problems. *Dev Psychol*. 2003;39(2):189-200. doi:10.1037/0012-1649.39.2.189

20. Masten AS, Neemann J, Andenas S. Life events and adjustment in adolescents: The significance of event independence, desirability, and chronicity. *J Res Adolesc*. 1994;4(1):71-97. doi:10.1207/s15327795jra0401_5

21. Farrell AD, Ampy LA, Meyer AL. Identification and assessment of problematic interpersonal situations for urban adolescents. *J Clin Child Psychol*. 1998;27(3):293-305. doi:10.1207/s15374424jccp2703_6

22. Petersen AC, Crockett L, Richards M, Boxer A. A self-report measure of pubertal status: Reliability, validity, and initial norms. *J Youth Adolesc*. 1988;17(2):117-133. doi:10.1007/BF01537962

23. Power JD, Barnes KA, Snyder AZ, Schlaggar BL, Petersen SE. Spurious but systematic correlations in functional connectivity MRI networks arise from subject motion. *NeuroImage*. 2012;59(3):2142-2154. doi:10.1016/j.neuroimage.2011.10.018

24. Hoaglin DC, Iglewicz B. Fine-Tuning Some Resistant Rules for Outlier Labeling. *J Am Stat Assoc*. 1987;82(400):1147-1149. doi:10.2307/2289392

© 2020 Goetschius LG et al. *JAMA Network Open.*

eTable 1. Participant Demographics

Included vs. Full Sample Comparison			
	**Included Sample (N=175)**	**Full Sample (N=237)**	**Statistically Different?**
**Age**	*M* = 15.88 yrs | *SD* = 0.53	*M* = 15.88 yrs | *SD* = 0.54	No. $t(379.27) = 0.10$, p=0.918
**Puberty**	*M* = 3.25 | *SD* = 0.58	*M* = 3.24 | *SD* = 0.59	No. $t(377.67) = 0.30$, p=0.770
**Gender**	F = 98 | M = 77	F = 125 | M = 112	No. $\chi^2(1) = 0.18$, $p = 0.670$
**Race**	African American: 127<br>Caucasian: 26<br>Other: 22	African American: 170<br>Caucasian: 34<br>Other: 33	No. $\chi^2(2) = 0.40$, $p = 0.820$
**Annual Income**	$4,999 or less: 22<br>$5,000 to $19,999: 29<br>$20,000 to $39,999: 50<br>$40,000 to $69,999: 33<br>$70,000 or more: 28<br>Not Report/Missing: 13	$4,999 or less: 28<br>$5,000 to $19,999: 41<br>$20,000 to $39,999: 66<br>$40,000 to $69,999: 46<br>$70,000 or more: 35<br>Not Report/Missing: 21	No. $\chi^2(5) = 0.34$, $p = 0.997$

© 2020 Goetschius LG et al. *JAMA Network Open.*

eTable 2

MNI coordinates for ROIs.

**Region**	**MNI Coordinates**	**Network**
**Left Hemisphere**		
Insula	-34 20 -4	Salience
Amygdala	-24 -6 -16	Salience
Dorsal Anterior Cingulate Cortex	0 46 6	Salience
Dorsolateral Prefrontal Cortex	-46 12 34	Salience
Inferior Parietal Lobule	-42 -52 48	Default Mode
Posterior Cingulate Cortex	-4 52 48	Default Mode
Medial Temporal Gyrus	-62 -26 -18	Default Mode
**Right Hemisphere**		
Insula	36 20 -4	Salience
Amygdala	24 -2 -16	Salience
Dorsal Anterior Cingulate Cortex	4 26 28	Salience
Dorsolateral Prefrontal Cortex	52 12 34	Salience
Inferior Parietal Lobule	46 -52 48	Default Mode
Posterior Cingulate Cortex	8 -52 28	Default Mode
Medial Temporal Gyrus	58 -16 20	Default Mode

Note: ROIs were selected based on their inclusion in either the Salience or Default Mode networks according to the NeuroSynth database (search terms were "Salience Network" and "Default Mode," respectively). Additionally, the MNI coordinates for all ROIs were preregistered with the Open Science Framework.

© 2020 Goetschius LG et al. *JAMA Network Open.*

eTable 3

Regression results for network density.

Predictor	*b*	*b* 95% CI [LL, UL]	*beta*	*beta* 95% CI [LL, UL]	*r*	Fit
**Total Density**						
(Intercept)	47.20	[38.30, 56.11]				
**Violence Exposure***	**-2.81**	**[-5.01, -0.60]**	**-0.23**	**[-0.41, -0.05]**	**-.13**	
Social Deprivation	1.51	[-0.63, 3.65]	0.12	[-0.05, 0.30]	.06	
**Motion [1]***	**13.24**	**[3.52, 22.96]**	**0.21**	**[0.06, 0.37]**	**.17**	
Gender	-2.22	[-4.65, 0.21]	-0.18	[-0.37, 0.02]	-.08	
Race_1[2]	0.90	[-2.17, 3.98]	0.06	[-0.15, 0.28]	-.08	
Race_2[2]	3.16	[-0.61, 6.94]	0.18	[-0.03, 0.39]	.16	
Pubertal Development	-1.37	[-3.44, 0.69]	-0.13	[-0.32, 0.06]	-.03	
Current Life Stress[3]	-0.08	[-0.24, 0.09]	-0.07	[-0.22, 0.08]	-.07	
Maternal Education[4]	-0.08	[-1.10, 0.93]	-0.01	[-0.18, 0.15]	.03	
Maternal Marital Status[4]	-0.29	[-2.80, 2.23]	-0.02	[-0.18, 0.15]	-.08	
						R^2 = .122
						95% CI[.00,.17]
**Salience Network Density**						
(Intercept)	17.37	[14.22, 20.52]				
**Violence Exposure***	**-1.01**	**[-1.79, -0.23]**	**-0.24**	**[-0.42, -0.05]**	**-.12**	
Social Deprivation	0.59	[-0.17, 1.34]	0.14	[-0.04, 0.32]	.06	
**Motion [1]***	**4.97**	**[1.54, 8.41]**	**0.23**	**[0.07, 0.39]**	**.20**	
Gender	-0.33	[-1.19, 0.53]	-0.07	[-0.27, 0.12]	-.01	
Race_1[2]	0.24	[-0.85, 1.33]	0.05	[-0.17, 0.26]	-.06	
Race_2[2]	0.78	[-0.56, 2.11]	0.12	[-0.09, 0.34]	.12	
Pubertal Development	-0.37	[-1.10, 0.36]	-0.10	[-0.29, 0.09]	-.07	
Current Life Stress[3]	-0.00	[-0.06, 0.06]	-0.01	[-0.16, 0.14]	-.03	
Maternal Education[4]	-0.05	[-0.40, 0.31]	-0.02	[-0.18, 0.14]	.02	
Maternal Marital Status[4]	-0.31	[-1.19, 0.58]	-0.06	[-0.22, 0.11]	-.09	
						R^2 = .106
						95% CI[.00,.15]
**Density Between Salience & Default Mode Networks**						
(Intercept)	18.74	[13.95, 23.53]				
**Violence Exposure***	**-1.33**	**[-2.51, -0.14]**	**-0.20**	**[-0.39, -0.02]**	**-.09**	
Social Deprivation	0.65	[-0.50, 1.80]	0.10	[-0.08, 0.28]	.06	
**Motion [1]***	**8.55**	**[3.33, 13.78]**	**0.26**	**[0.10, 0.41]**	**.22**	
Gender	-1.07	[-2.37, 0.24]	-0.16	[-0.35, 0.04]	-.04	

© 2020 Goetschius LG et al. *JAMA Network Open.*

Race_1[2]	0.28	[-1.37, 1.94]	0.04	[-0.18, 0.25]	-.07	
Race_2[2]	1.47	[-0.56, 3.51]	0.15	[-0.06, 0.37]	.14	
Pubertal Development	-0.79	[-1.90, 0.33]	-0.13	[-0.32, 0.06]	-.07	
Current Life Stress[3]	-0.05	[-0.14, 0.04]	-0.08	[-0.23, 0.07]	-.09	
Maternal Education[4]	-0.01	[-0.56, 0.54]	-0.00	[-0.17, 0.16]	.01	
Maternal Marital Status[4]	0.40	[-0.95, 1.75]	0.05	[-0.12, 0.21]	-.01	
						R^2 = .122<br>95% CI[.00,.17]
**Default Mode Network Density**						
(Intercept)	11.09	[8.18, 14.01]				
Violence Exposure	-0.47	[-1.19, 0.25]	-0.12	[-0.31, 0.06]	-.12	
Social Deprivation	0.27	[-0.43, 0.97]	0.07	[-0.11, 0.25]	.01	
Motion [1]	-0.29	[-3.46, 2.89]	-0.01	[-0.17, 0.15]	-.06	
Gender*	-0.82	[-1.62, -0.03]	-0.20	[-0.40, -0.01]	-.19	
Race_1[2]	0.38	[-0.62, 1.39]	0.08	[-0.14, 0.30]	-.07	
Race_2[2]	0.91	[-0.32, 2.15]	0.16	[-0.06, 0.38]	.15	
Pubertal Development	-0.22	[-0.89, 0.46]	-0.06	[-0.26, 0.13]	.09	
Current Life Stress[3]	-0.02	[-0.08, 0.03]	-0.06	[-0.22, 0.09]	-.05	
Maternal Education[4]	-0.03	[-0.36, 0.31]	-0.01	[-0.18, 0.15]	.05	
Maternal Marital Status[4]	-0.38	[-1.20, 0.44]	-0.08	[-0.25, 0.09]	-.13	
						R^2 = .079
						95% CI[.00,.11]

Note. A significant *b*-weight indicates the beta-weight and semi-partial correlation are also significant. *b* represents unstandardized regression weights. *beta* indicates the standardized regression weights. *r* represents the zero-order correlation. *LL* and *UL* indicate the lower and upper limits of a confidence interval, respectively.
* indicates significant predictor of network density
[1]Motion is measured using mean relative framewise displacement
[2]Dummy coded variables represented 3 category race variable (African American, Caucasian, Other)
[3]Current life stress is measured using the Adolescent Life Events Scale
[4]Maternal variable at the child's birth

© 2020 Goetschius LG et al. *JAMA Network Open.*

eTable 4

Node degree for ROIs that were not significantly associated with violence exposure or social deprivation using the Bonferroni-corrected significance threshold.

Predictor	*b*	*b* 95% CI [LL, UL]	*beta*	*beta* 95% CI [LL, UL]	*r*	Fit
**Left Amygdala Degree**						
(Intercept)	2.87	[2.55, 3.20]				
Violence Exposure	-0.60	[-1.13, -0.07]	-0.19	[-0.36, -0.02]	-.04	
Social Deprivation	0.30	[-0.23, 0.82]	0.09	[-0.07, 0.26]	.07	
Motion [1]	6.04	[3.69, 8.40]	0.37	[0.23, 0.52]	.34	
						R^2 = .143
						95% CI[.05,.23]
**Left Dorsal Anterior Cingulate Cortex Degree**						
(Intercept)	6.47	[6.15, 6.79]				
Violence Exposure	-0.35	[-0.87, 0.17]	-0.12	[-0.30, 0.06]	-.08	
Social Deprivation	0.18	[-0.34, 0.69]	0.06	[-0.12, 0.24]	.01	
Motion [1]	0.61	[-1.70, 2.92]	0.04	[-0.11, 0.20]	.02	
						R^2 = .011
						95% CI[.00,.04]
**Left Dorsolateral Prefrontal Cortex Degree**						
(Intercept)	6.96	[6.53, 7.40]				
Violence Exposure	-0.62	[-1.33, 0.08]	-0.16	[-0.34, 0.02]	-.12	
Social Deprivation	0.34	[-0.36, 1.04]	0.09	[-0.09, 0.26]	-.00	
Motion [1]	-0.57	[-3.71, 2.56]	-0.03	[-0.18, 0.13]	-.05	
						R^2 = .020
						95% CI[.00,.06]
**Left Anterior Insula Degree**						
(Intercept)	6.02	[5.76, 6.28]				
Violence Exposure	-0.50	[-0.93, -0.08]	-0.21	[-0.39, -0.03]	-.12	
Social Deprivation	0.26	[-0.17, 0.68]	0.11	[-0.07, 0.28]	.02	
Motion [1]	1.38	[-0.52, 3.27]	0.11	[-0.04, 0.27]	.08	
						R^2 = .037
						95% CI[.00,.09]
**Left Medial Temporal Gyrus Degree**						
(Intercept)	6.03	[5.62, 6.45]				
Violence Exposure	-0.55	[-1.21, 0.12]	-0.15	[-0.32, 0.03]	-.06	
Social Deprivation	0.65	[-0.01, 1.31]	0.17	[-0.00, 0.35]	.09	
Motion [1]	-0.68	[-3.65, 2.29]	-0.04	[-0.19, 0.12]	-.04	
						R^2 = .026
						95% CI[.00,.07]
**Left Posterior Cingulate Cortex Degree**						
(Intercept)	8.40	[8.03, 8.76]				

© 2020 Goetschius LG et al. *JAMA Network Open.*

Violence Exposure	-0.61	[-1.21, -0.01]	-0.18	[-0.36, -0.00]	-.11	
Social Deprivation	0.35	[-0.24, 0.94]	0.11	[-0.07, 0.28]	.02	
Motion [1]	0.78	[-1.87, 3.44]	0.05	[-0.11, 0.20]	.02	
						R^2 = .024
						95% CI[.00,.07]
**Right Amygdala Degree**						
(Intercept)	4.05	[3.80, 4.29]				
Violence Exposure	0.00	[-0.40, 0.40]	0.00	[-0.16, 0.16]	.13	
Social Deprivation	0.05	[-0.34, 0.45]	0.02	[-0.14, 0.18]	.11	
Motion [1]	5.38	[3.60, 7.16]	0.43	[0.29, 0.57]	.43	
						R^2 = .188
						95% CI[.08,.28]
**Right Dorsal Anterior Cingulate Cortex Degree**						
(Intercept)	7.39	[7.13, 7.64]				
Violence Exposure	-0.40	[-0.81, 0.01]	-0.17	[-0.35, 0.00]	-.08	
Social Deprivation	0.12	[-0.28, 0.53]	0.05	[-0.12, 0.23]	.02	
Motion [1]	3.01	[1.19, 4.83]	0.25	[0.10, 0.40]	.22	
						R^2 = .069
						95% CI[.00,.14]
**Right Dorsolateral Prefrontal Cortex Degree**						
(Intercept)	6.38	[6.08, 6.68]				
Violence Exposure	-0.39	[-0.87, 0.09]	-0.14	[-0.32, 0.03]	-.03	
Social Deprivation	0.40	[-0.08, 0.88]	0.15	[-0.03, 0.32]	.10	
Motion [1]	1.71	[-0.45, 3.86]	0.12	[-0.03, 0.28]	.12	
						R^2 = .033
						95% CI[.00,.09]
**Right Inferior Parietal Lobule Degree**						
(Intercept)	5.45	[5.15, 5.75]				
Violence Exposure	-0.49	[-0.97, 0.00]	-0.18	[-0.36, 0.00]	-.16	
Social Deprivation	0.06	[-0.42, 0.54]	0.02	[-0.15, 0.20]	-.07	
Motion [1]	0.43	[-1.74, 2.59]	0.03	[-0.12, 0.19]	-.01	
						R^2 = .026
						95% CI[.00,.08]
**Right Medial Temporal Gyrus Degree**						
(Intercept)	4.69	[4.42, 4.96]				
Violence Exposure	0.14	[-0.30, 0.57]	0.05	[-0.12, 0.22]	.18	
Social Deprivation	0.26	[-0.17, 0.69]	0.10	[-0.07, 0.27]	.19	
Motion [1]	3.46	[1.53, 5.39]	0.27	[0.12, 0.41]	.30	
						R^2 = .11
						95% CI[.03,.19]
**Right Posterior Cingulate Cortex Degree**						
(Intercept)	3.99	[3.67, 4.30]				
Violence Exposure	-0.29	[-0.80, 0.22]	-0.10	[-0.28, 0.08]	-.12	
Social Deprivation	-0.25	[-0.76, 0.25]	-0.09	[-0.26, 0.09]	-.11	
Motion [1]	1.88	[-0.39, 4.15]	0.13	[-0.03, 0.28]	.08	

© 2020 Goetschius LG et al. *JAMA Network Open.*

R^2 = .032
95% CI[.00,.09]
Note. A significant *b*-weight indicates the beta-weight and semi-partial correlation are also significant. *b* represents unstandardized regression weights. *beta* indicates the standardized regression weights. *r* represents the zero-order correlation. *LL* and *UL* indicate the lower and upper limits of a confidence interval, respectively.<br>*Significant at a Bonferroni-corrected threshold of p = 0.004 (p=0.05/14 tests)<br>[1]Motion is measured using mean relative framewise displacement

© 2020 Goetschius LG et al. *JAMA Network Open.*

eTable 5
Node density adjusted for covariates

Predictor	*b*	*b* 95% CI [LL, UL]	*beta*	*beta* 95% CI [LL, UL]	*r*	Fit
**Left Inferior Parietal Lobule**						
(Intercept)	9.14	[6.79, 11.49]				
**Violence Exposure***	**-0.75**	**[-1.33, -0.17]**	**-0.23**	**[-0.42, -0.05]**	**-.17**	
Social Deprivation	0.36	[-0.20, 0.93]	0.11	[-0.06, 0.29]	.03	
Motion [1]	1.27	[-1.29, 3.84]	0.08	[-0.08, 0.24]	.03	
Gender	-0.04	[-0.09, 0.00]	-0.15	[-0.30, 0.01]	-.13	
Race_1[2]	-0.37	[-0.91, 0.18]	-0.13	[-0.32, 0.06]	-.09	
Race_2[2]	-0.71	[-1.36, -0.07]	-0.21	[-0.41, -0.02]	.17	
Pubertal Development	0.30	[-0.51, 1.11]	0.08	[-0.14, 0.29]	.03	
Current Life Stress[3]	0.85	[-0.15, 1.84]	0.18	[-0.03, 0.39]	-.14	
Maternal Education[4]	-0.09	[-0.35, 0.18]	-0.05	[-0.22, 0.11]	.00	
Maternal Marital Status[4]	-0.09	[-0.75, 0.57]	-0.02	[-0.19, 0.14]	-.07	
						R^2 = .117
						95% CI[.00,.16]
**Right Insula**						
(Intercept)	8.44	[6.83, 10.06]				
**Violence Exposure***	**-0.64**	**[-1.04, -0.24]**	**-0.28**	**[-0.46, -0.11]**	**-.17**	
Social Deprivation	0.4	[0.06, 0.84]	0.20	[0.03, 0.37]	.06	
Motion [1]	1.28	[-0.48, 3.04]	0.11	[-0.04, 0.27]	.08	
Gender	-0.00	[-0.03, 0.03]	-0.02	[-0.17, 0.13]	-.05	
Race_1[2]	-0.36	[-0.73, 0.02]	-0.18	[-0.36, 0.01]	-.10	
Race_2[2]	-0.26	[-0.70, 0.19]	-0.11	[-0.30, 0.08]	-.01	
Pubertal Development	0.01	[-0.54, 0.57]	0.01	[-0.21, 0.22]	-.14	
Current Life Stress[3]	0.58	[-0.11, 1.27]	0.18	[-0.03, 0.39]	.21	
Maternal Education[4]	0.17	[-0.01, 0.36]	0.15	[-0.01, 0.31]	.17	
Maternal Marital Status[4]	0.15	[-0.30, 0.61]	0.05	[-0.11, 0.22]	-.07	
						R^2 = .149
						95% CI[.02,.20]

Note. A significant *b*-weight indicates the beta-weight and semi-partial correlation are also significant. *b* represents unstandardized regression weights. *beta* indicates the standardized regression weights. *r* represents the zero-order correlation. *LL* and *UL* indicate the lower and upper limits of a confidence interval, respectively.
*Significant predictor of node degree adjusting for covariates.
[1]Motion is measured using mean relative framewise displacement
[2]Dummy coded variables represented 3 category race variable (African American, Caucasian, Other)
[3]Current life stress is measured using the Adolescent Life Events Scale
[4]Maternal variable at the child's birth

© 2020 Goetschius LG et al. *JAMA Network Open.*

eTable 6

Logistic regression results adjusted for covariates

Predictor	*b*	*SE*	*Odds Ratio*	*Odds Ratio* 95% CI [LL, UL]
(Intercept)	-0.20	1.82	0.82	[0.02, 32.70]
**Violence Exposure**	**0.93**	**0.54**	**2.54**	**[0.92, 7.87]**
Social Deprivation	-0.38	0.47	0.68	[0.27, 1.77]
**Motion [1]**	**7.32**	**3.54**	**1516.93**	**[3.58, 4284250.00]**
Gender	0.57	0.50	1.77	[0.67, 4.77]
Race_1[2]	-0.58	0.71	0.56	[0.11, 2.02]
Race_2[2]	-1.13	0.80	0.32	[0.06, 1.46]
Pubertal Development	0.14	0.41	1.16	[0.50, 2.59]
Current Life Stress[3]	0.05	0.04	1.06	[0.98, 1.14]
Maternal Education[4]	0.07	0.20	1.07	[0.72, 1.61]
Maternal Marital Status[4]	0.05	0.49	1.05	[0.39, 2.72]

[1]Motion is measured using mean relative framewise displacement
[2]Dummy coded variables represented 3 category race variable (African American, Caucasian, Other)
[3]Current life stress is measured using the Adolescent Life Events Scale
[4]Maternal variable at the child's birth

© 2020 Goetschius LG et al. *JAMA Network Open.*

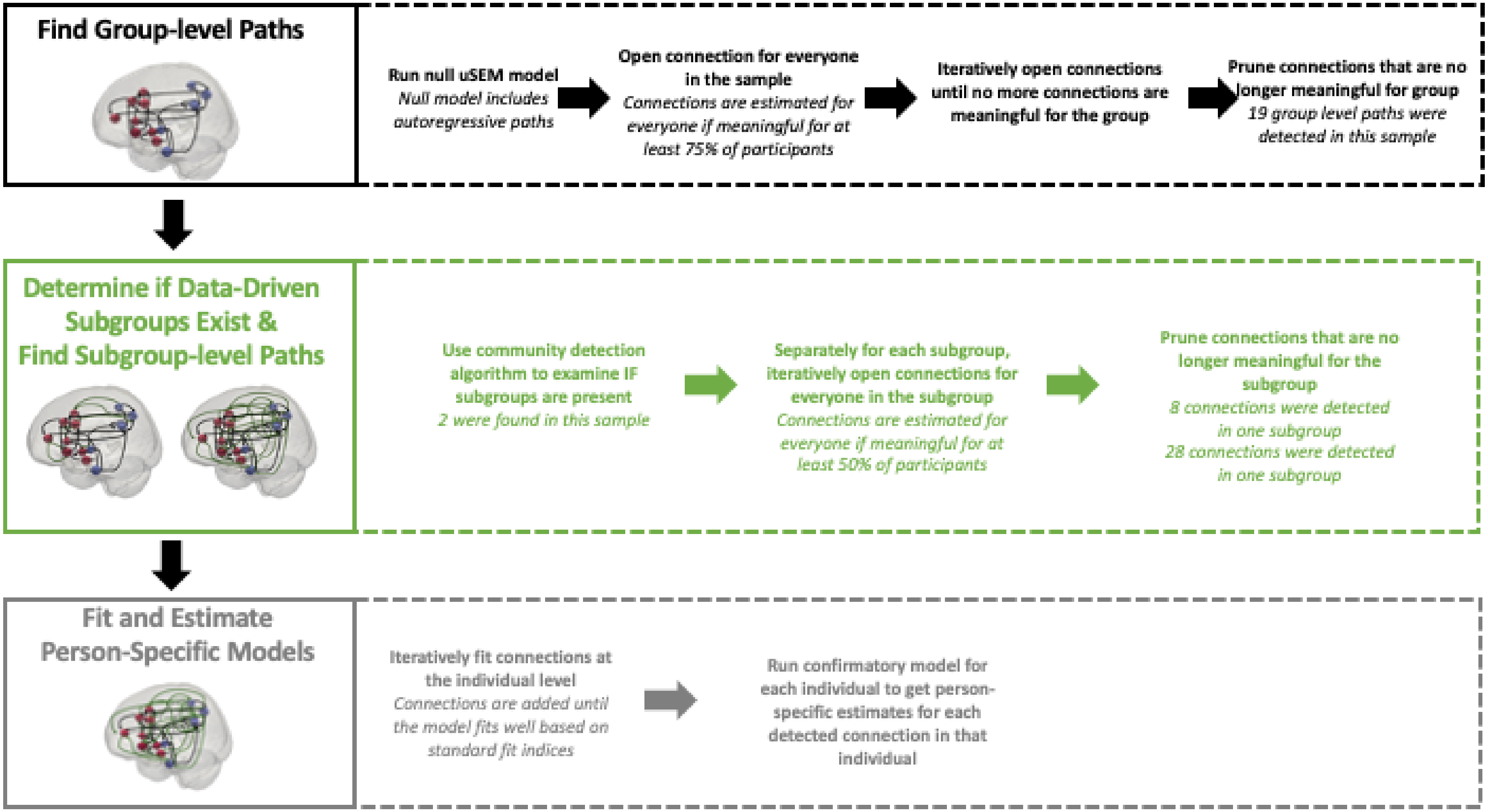

eFigure 1. Flowchart of the S-GIMME analytical steps

© 2020 Goetschius LG et al. *JAMA Network Open.*

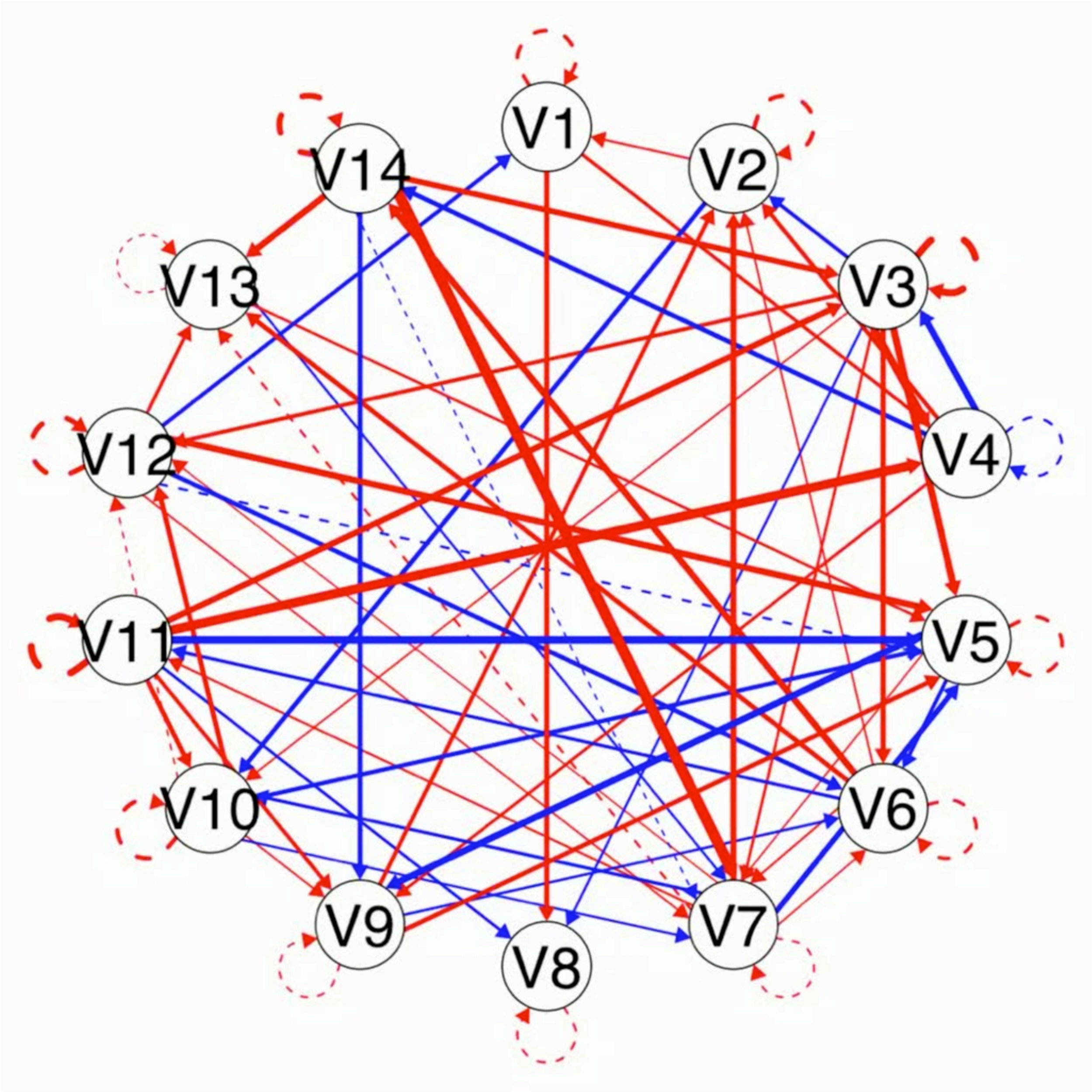

eFigure 2: Individual connectivity maps. Red connections have positive beta weights, blue connections have negative beta weights. Solid connections are contemporaneous and dashed connections are lagged. V1: left amygdala, V2: left dorsal anterior cingulate cortex (dACC), V3: dorsolateral prefrontal cortex (dlPFC), V4: left insula, V5: left inferior parietal lobule (IPL), V6: left medial temporal gyrus (MTG), V7: left posterior cingulate cortex, V8: right amygdala, V9: right dACC, V10: right dlPFC, V11: right insula, V12: right IPL, V13: right MTG, V14: right PCC. Video also included separately.

© 2020 Goetschius LG et al. *JAMA Network Open.*

eTable 7. Model fit for each participant. Connections were not added to individual models when 2 out of the 4 fit indices met the following thresholds: RMSEA ≤0.05; SRMR ≤0.05; CFI ≥0.95; NNFI ≥0.95.

	χ^2	df	npar	RMSEA	SRMR	NNFI	CFI
1	337.464	215	219	0.0493	0.0498	0.9468	0.9698
2	460.4917	232	202	0.0649	0.0434	0.919	0.9503
3	442.6814	227	207	0.0637	0.0493	0.9176	0.9505
4	369.3483	230	204	0.0509	0.0497	0.9395	0.9632
5	356.2464	224	210	0.0502	0.0499	0.9424	0.9658
6	422.89	230	204	0.0599	0.0472	0.9367	0.9615
7	400.1407	228	206	0.0568	0.0469	0.9183	0.9507
8	395.4893	229	205	0.0557	0.0493	0.9342	0.9602
9	472.9349	228	206	0.0678	0.0485	0.9261	0.9554
10	349.8288	222	212	0.0496	0.0511	0.9436	0.9668
11	446.6057	229	205	0.0637	0.0492	0.9183	0.9505
12	505.731	218	216	0.0751	0.0491	0.9157	0.9514
13	417.9443	221	213	0.0617	0.0485	0.9263	0.9569
14	451.117	227	207	0.065	0.049	0.9287	0.9572
15	405.1967	229	205	0.0573	0.0486	0.9292	0.9571
16	377.3698	228	206	0.0529	0.0486	0.952	0.971
17	375.7425	200	234	0.0613	0.0485	0.9211	0.9583
18	418.1664	219	215	0.0623	0.0482	0.9139	0.9501
19	362.0274	218	216	0.0531	0.0485	0.9154	0.9512
20	489.3867	233	201	0.0686	0.0484	0.9203	0.9509
21	355.5109	230	204	0.0483	0.0547	0.9471	0.9678
22	381.252	231	203	0.0527	0.0492	0.9311	0.9579
23	359.1018	209	225	0.0554	0.0499	0.9229	0.9574
24	366.8721	232	202	0.0498	0.0526	0.9266	0.9549
25	422.4284	226	208	0.0609	0.0466	0.9175	0.9507
26	482.5072	234	200	0.0674	0.0461	0.9201	0.9505
27	357.8082	220	214	0.0517	0.0498	0.935	0.9621

© 2020 Goetschius LG et al. *JAMA Network Open.*

28	441.1115	227	207	0.0635	0.0455	0.9184	0.951
29	415.5675	213	221	0.0638	0.0496	0.9249	0.9577
30	435.3762	225	209	0.0632	0.0469	0.9164	0.9502
31	459.8657	216	218	0.0695	0.049	0.9279	0.9588
32	404.2475	215	219	0.0613	0.0476	0.9241	0.9569
33	363.5178	222	212	0.0522	0.0446	0.9168	0.9511
34	394.4465	217	217	0.0591	0.0498	0.9381	0.9645
35	512.738	221	213	0.0751	0.0461	0.9164	0.9511
36	419.0826	227	207	0.0601	0.0496	0.9189	0.9513
37	391.933	225	209	0.0563	0.0438	0.9204	0.9526
38	355.2865	226	208	0.0494	0.053	0.9259	0.9557
39	431.6301	221	213	0.0638	0.0487	0.9246	0.9559
40	363.5577	232	202	0.0492	0.0502	0.9342	0.9596
41	418.9789	216	218	0.0634	0.0494	0.9146	0.9512
42	370.4016	221	213	0.0537	0.0496	0.9307	0.9595
43	357.6225	219	215	0.052	0.0497	0.9424	0.9666
44	364.269	218	216	0.0535	0.05	0.9169	0.9521
45	404.6379	213	221	0.062	0.048	0.9374	0.9647
46	481.5645	215	219	0.0728	0.0443	0.9148	0.9515
47	360.3157	225	209	0.0507	0.0493	0.9179	0.9511
48	435.7415	217	217	0.0656	0.045	0.9164	0.952
49	387.1817	225	209	0.0555	0.0459	0.9177	0.951
50	428.6833	229	205	0.061	0.0489	0.9352	0.9608
51	414.8962	233	201	0.0578	0.0424	0.9191	0.9501
52	395.9013	212	222	0.0609	0.0447	0.9125	0.9509
53	532.6246	221	213	0.0776	0.047	0.9148	0.9502
54	416.5936	221	213	0.0615	0.0434	0.9152	0.9504
55	434.5957	208	226	0.0682	0.0497	0.9131	0.9522
56	418.6393	236	198	0.0575	0.0485	0.9256	0.9535
57	351.4741	222	212	0.0499	0.0499	0.9385	0.9639
58	398.2812	236	198	0.0542	0.0476	0.9511	0.9695
59	355.4745	228	206	0.0489	0.0483	0.922	0.953

© 2020 Goetschius LG et al. *JAMA Network Open.*

60	471.3257	231	203	0.0667	0.0487	0.9262	0.9549
61	343.2778	206	228	0.0534	0.0495	0.9509	0.9732
62	431.6664	210	224	0.0672	0.0408	0.915	0.9528
63	432.934	225	209	0.0628	0.0465	0.9186	0.9516
64	341.8362	219	215	0.049	0.0512	0.9526	0.9726
65	395.5717	226	208	0.0566	0.0472	0.9167	0.9502
66	445.6803	225	209	0.0647	0.05	0.9164	0.9502
67	481.0292	219	215	0.0715	0.0441	0.9148	0.9506
68	388.5093	217	217	0.0581	0.0445	0.9165	0.9521
69	385.9336	210	224	0.0598	0.0411	0.9142	0.9524
70	422.1714	227	207	0.0606	0.0487	0.9353	0.9612
71	372.7023	236	198	0.0498	0.0488	0.9441	0.9651
72	435.3817	223	211	0.0638	0.0498	0.9187	0.952
73	368.3488	216	218	0.0549	0.0491	0.9404	0.9659
74	379.8688	207	227	0.0597	0.0478	0.9274	0.9602
75	460.2895	214	220	0.0701	0.0496	0.9288	0.9597
76	524.4858	217	217	0.0778	0.0436	0.9139	0.9506
77	356.5818	230	204	0.0485	0.057	0.9345	0.9602
78	428.9118	232	202	0.0602	0.0498	0.9278	0.9557
79	424.6102	226	208	0.0613	0.0497	0.9229	0.9539
80	404.0907	216	218	0.061	0.0439	0.9171	0.9526
81	408.21	214	220	0.0623	0.0496	0.9218	0.9557
82	337.0235	216	218	0.0489	0.052	0.9491	0.9709
83	416.4031	218	216	0.0624	0.0437	0.9179	0.9527
84	473.7268	228	206	0.0679	0.0442	0.919	0.9511
85	388.1109	233	201	0.0533	0.0494	0.9487	0.9684
86	378.0401	221	213	0.0551	0.0492	0.9245	0.9559
87	472.1856	218	216	0.0706	0.0499	0.9155	0.9512
88	417.637	233	201	0.0582	0.0498	0.9384	0.962
89	383.6733	230	204	0.0534	0.0481	0.9293	0.957
90	397.4964	222	212	0.0581	0.0476	0.9174	0.9515
91	358.8544	229	205	0.0492	0.0538	0.9407	0.9641

© 2020 Goetschius LG et al. *JAMA Network Open.*

92	515.0297	237	197	0.0708	0.0479	0.9228	0.9516
93	339.9077	222	212	0.0476	0.0624	0.9418	0.9658
94	408.5504	209	225	0.0639	0.041	0.9114	0.951
95	549.3469	226	208	0.0782	0.0464	0.9165	0.9501
96	376.8911	225	209	0.0537	0.0492	0.9351	0.9614
97	440.472	221	213	0.0651	0.0471	0.9172	0.9516
98	347.1012	222	212	0.0491	0.0489	0.948	0.9695
99	381.2555	224	210	0.0548	0.048	0.9167	0.9506
100	441.3668	230	204	0.0627	0.0499	0.925	0.9543
101	407.0288	221	213	0.06	0.0495	0.9222	0.9545
102	376.2155	216	218	0.0563	0.0464	0.9131	0.9503
103	366.2102	223	211	0.0524	0.0497	0.9416	0.9656
104	381.601	233	201	0.0522	0.0478	0.9242	0.9533
105	457.7367	235	199	0.0636	0.048	0.9206	0.9506
106	423.4817	214	220	0.0647	0.0492	0.9321	0.9616
107	433.4452	227	207	0.0623	0.0468	0.92	0.952
108	449.1592	228	206	0.0644	0.0496	0.9176	0.9503
109	477.1445	227	207	0.0686	0.0496	0.9192	0.9515
110	412.928	223	211	0.0603	0.0486	0.9183	0.9518
111	395.8876	234	200	0.0544	0.0491	0.9222	0.9519
112	509.8378	202	232	0.0807	0.0494	0.9094	0.9516
113	379.6696	224	210	0.0545	0.0485	0.9436	0.9666
114	393.9832	229	205	0.0555	0.0488	0.9277	0.9562
115	420.1125	231	203	0.0591	0.0487	0.9283	0.9562
116	461.147	216	218	0.0696	0.0436	0.9167	0.9524
117	435.54	218	216	0.0653	0.047	0.9172	0.9522
118	400.423	221	213	0.0589	0.0478	0.9294	0.9587
119	405.4778	214	220	0.0618	0.0485	0.9135	0.951
120	435.3515	229	205	0.0621	0.0492	0.9258	0.9551
121	467.4572	219	215	0.0696	0.0495	0.9162	0.9515
122	451.823	226	208	0.0653	0.0457	0.9193	0.9517
123	444.5608	213	221	0.0682	0.0471	0.9164	0.9529

© 2020 Goetschius LG et al. *JAMA Network Open.*

124	360.8171	216	218	0.0535	0.0495	0.9209	0.9548
125	428.2471	220	214	0.0636	0.044	0.9173	0.9519
126	371.5123	213	221	0.0564	0.0456	0.928	0.9594
127	374.1864	232	202	0.0512	0.0453	0.9215	0.9518
128	410.7942	212	222	0.0633	0.0487	0.9291	0.9602
129	480.7249	220	214	0.0712	0.048	0.9154	0.9507
130	408.7257	224	210	0.0594	0.0437	0.9166	0.9506
131	473.8235	209	225	0.0736	0.0416	0.9114	0.951
132	344.6724	219	215	0.0495	0.0532	0.9407	0.9656
133	430.6263	230	204	0.0611	0.0464	0.9211	0.952
134	432.7567	231	203	0.0611	0.0487	0.9241	0.9536
135	511.666	240	194	0.0696	0.0456	0.9231	0.9512
136	435.3927	222	212	0.0641	0.0482	0.9176	0.9516
137	360.0409	216	218	0.0534	0.0489	0.9322	0.9613
138	390.6012	230	204	0.0546	0.0444	0.9307	0.9578
139	411.2425	233	201	0.0572	0.0482	0.9352	0.9601
140	441.3396	221	213	0.0653	0.049	0.9171	0.9515
141	423.1329	221	213	0.0625	0.0455	0.9182	0.9522
142	484.8995	218	216	0.0723	0.0422	0.9205	0.9542
143	424.9599	220	214	0.0631	0.0485	0.9283	0.9583
144	437.5468	233	201	0.0613	0.0492	0.9285	0.9559
145	395.4005	228	206	0.056	0.0491	0.9282	0.9567
146	430.6302	229	205	0.0613	0.0475	0.9205	0.9518
147	505.7797	230	204	0.0716	0.0463	0.9206	0.9517
148	380.6049	215	219	0.0574	0.0444	0.9155	0.9519
149	481.3911	226	208	0.0695	0.0499	0.9295	0.9579
150	361.4001	220	214	0.0524	0.0465	0.9516	0.9718
151	356.5355	227	207	0.0494	0.0513	0.9247	0.9548
152	395.1945	227	207	0.0563	0.0483	0.9302	0.9581
153	420.3603	223	211	0.0615	0.0499	0.9183	0.9518
154	395.4	226	208	0.0566	0.0472	0.9178	0.9508
155	422.053	233	201	0.0589	0.05	0.9216	0.9517

© 2020 Goetschius LG et al. *JAMA Network Open.*

156	361.1325	224	210	0.0511	0.0497	0.9329	0.9602
157	443.7209	233	201	0.0622	0.0493	0.9204	0.9509
158	419.7464	215	219	0.0638	0.0496	0.9331	0.962
159	422.6659	226	208	0.061	0.0489	0.917	0.9504
160	431.8286	228	206	0.0618	0.047	0.9181	0.9506
161	456.3976	235	199	0.0635	0.0475	0.921	0.9509
162	348.7459	222	212	0.0494	0.0498	0.9431	0.9666
163	384.086	226	208	0.0547	0.0487	0.9244	0.9548
164	450.2674	222	212	0.0663	0.0499	0.928	0.9577
165	333.3395	216	218	0.0482	0.0516	0.932	0.9612
166	418.5673	229	205	0.0595	0.0492	0.9204	0.9518
167	398.5382	218	216	0.0595	0.0486	0.9348	0.9624
168	353.7505	225	209	0.0495	0.0509	0.9235	0.9545
169	415.5257	230	204	0.0587	0.0484	0.9317	0.9584
170	402.5641	223	211	0.0587	0.0462	0.9209	0.9534
171	384.9787	229	205	0.054	0.0478	0.939	0.963
172	403.9139	215	219	0.0613	0.0494	0.9161	0.9523
173	374.1356	226	208	0.0529	0.0491	0.9294	0.9578
174	390.1415	216	218	0.0587	0.0488	0.935	0.9628
175	434.5492	215	219	0.0661	0.048	0.9153	0.9518

© 2020 Goetschius LG et al. *JAMA Network Open.*
